# Genome-Wide Mapping of Yeast Histone Chaperone Anti-Silencing Function 1 Reveals Its Role in Condensin Binding with Chromatin

**DOI:** 10.1371/journal.pone.0108652

**Published:** 2014-09-29

**Authors:** Pooran Singh Dewari, Purnima Bhargava

**Affiliations:** Centre for Cellular and Molecular Biology (Council of Scientific and Industrial Research), Hyderabad, Andhra Pradesh, India; Newcastle University, United Kingdom

## Abstract

Genome-wide participation and importance of the histone chaperone Asf1 (Anti-Silencing Function 1) in diverse DNA transactions like replication, repair, heterochromatic silencing and transcription are well documented. Yet its genome-wide targets have not been reported. Using ChIP-seq method, we found that yeast Asf1 associates with 590 unique targets including centromeres, telomeres and condensin-binding sites. It is found selectively on highly transcribed regions, which include replication fork pause sites. Asf1 preferentially associates with the genes transcribed by RNA polymerase (pol) III where its presence affects RNA production and replication-independent histone exchange. On pol II-transcribed genes, a negative correlation is found between Asf1 and nucleosome occupancy. It is not enriched on most of the reported sites of histone exchange or on the genes, which are misregulated in the *asf1*Δ cells. Interestingly, chromosome-wide distributions of Asf1 and one of the condensin subunits, Brn1 show a nearly identical pattern. Moreover, Brn1 shows reduced occupancy at various condensin-binding sites in *asf1*Δ cells. These results along with high association of Asf1 with heterochromatic centromeres and telomeres ascribe novel roles to Asf1 in condensin loading and chromatin dynamics.

## Introduction

Organization of the eukaryotic genome into chromatin enables its compaction inside the cell nucleus and concomitant regulation of DNA-related processes [1]. Several mechanisms including histone modifications and ATP-dependent chromatin-remodeling, culminate into an altered chromatin structure [2], , which renders the *cis*-acting sites on the DNA accessible to the *trans*-acting factors. They often involve localized chromatin assembly/disassembly via eviction/deposition of the histones by specific histone chaperones, which bind dimers of canonical or variant histones H2A/H2B or H3/H4 [4], [5]. Histone chaperones also assist DNA transactions by exchanging old histones with new ones [6] and play important roles in replication and repair processes [7]. Anti-Silencing Function 1 (Asf1) is a highly conserved histone chaperone, which assists essentially all aspects of chromatin biology, including genome silencing by evicting/depositing H3/H4 dimers [8]. It participates in regulating histone synthesis [9], maintains supply of histones, interacts with DNA replication machinery at active replication forks, helps progression of replication fork and maintains replisome integrity [10]–[12]. It cooperates with other chaperones like CAF1, HIR, FACT and several other histone-binding factors in replication-coupled or replication-independent chromatin assembly [13]–[16].

Yeast cells lacking *ASF1* are sensitive to DNA damaging agents, as it plays important role in checkpoint signaling and genomic stability [13], [17], [18]. In addition to its role as chromatin assembly and disassembly factor, Asf1 is essentially required, in collaboration with histone acetyl-transferases (HATs), for the acetylation of lysine residues of histone H3 at positions 9 and 56 [19], [20]. Several studies have shown that chromatin reassembly and deactivation of damage checkpoint after double-strand break (DSB) repair require cooperative acetylation of H3K56 by the histone acetylase Rtt109 and Asf1 [21]–[24]. Deletion of Asf1 causes up-regulation as well as down-regulation of genes suggesting its role in transcriptional repression as well as activation in a context-dependent manner [25]–[28]. Association of Asf1 with active genes promotes histone eviction and deposition, thus facilitating transcription elongation by the RNA polymerase (pol) II [29], [30]. Studies in the budding yeast have shown replication-independent exchange of histone H3 throughout the genome [31]–[34]. Outside of the S phase, H3 exchange at promoters is higher than coding regions of the actively transcribed genes and requires Asf1 [32]–[34].

As evident from the above account, significant advances have been made in understanding the functional diversity of Asf1. However, despite genome-wide functional studies, its genome-wide association map in the budding yeast is not reported yet. Our high-resolution mapping of Asf1 along the 16 chromosomes of the budding yeast revealed that Asf1 specifically associates with highly active pol II and pol III-transcribed loci, which are also known sites of replication fork arrest [35], [36]. Its absence on most of the known sites of Asf1-dependent histone exchange or transcription, suggests its functions at a genomic locus do not depend on stable association with the site. The highest levels at centromeres as compared to telomeres and co-localization with condensin subunit Brn1 ascribe novel roles to Asf1.

## Materials and Methods

### Yeast strains, plasmids, growth conditions and antibodies

List of the yeast strains and sequences of the primers used in this study are given under the Tables S1 and S2 respectively. Unless otherwise stated, yeast cells were grown in YEP+2% glucose (YPD) medium to an OD_600_ of 0.7. Chromatin Immunoprecipitation (ChIP)-grade anti-HA was from Millipore; anti-Myc, anti-GFP and anti-HA antibodies were from Santa Cruz Biotechnology.

### ChIP, Real Time PCR and ChIP-sequencing

Binding of a histone chaperones to chromatin can be ubiquitous. Difficulty in cross-linking Asf1 to chromatin [37] has been one of the reasons that its genome-wide map has been elusive. We used a yeast strain expressing *ASF1*-18myc from its genomic locus (gift from Kevin Struhl, [30]) and prepared ChIP DNA samples as previously described [38], [39], except that cells were cross-linked with 1% formaldehyde for six hours [40] and chromatin was fragmented to mean size of 200–400 bp, using Bioruptor (Diagenode). The quality and size of the sheared DNA was checked by gel electrophoresis (Figure S1A). Purified DNA was either used for quantifying the amplicons (Figure S1B) by Real Time quantitative PCR or library preparation for sequencing with Illumina Genome Analyzer II, as per manufacturer's instructions. High-quality sequencing reads (phred score >20) were aligned, reporting unique and best alignment for each read, to budding yeast genome version sacCer 3. ChIP-seq data analysis was made according to our previously published method [41], as detailed further in the Supporting Information (Methods S1). HOMER package was used to make ready-to-visualize bedgraph files and peak calling. For each chromosomal feature, Asf1 tag count was calculated 1000 bp both upstream and downstream of genes and aligned according to TSS or TTS, using a 10 bp window and an overlap of 2 bp. Heat-maps were created using MultiExperiment Viewer (MeV).

### Histone exchange assay

As detailed in the Supplementary Information, yeast cells carrying a plasmid that contains *GAL1*-driven H3-(HA)_3_ gene were used for histone exchange assay [34]. Though the method used does not involve balanced expression of H3/H4, it does not influence the results obtained [33]. To calculate H3 exchange at Asf1-targeted loci, genome-wide histone H3 exchange data were extracted from a previous report [33] and analysed.

## Results

### Asf1 associates with the highly transcribed genes and the heterochromatic regions

**Figure 1:**
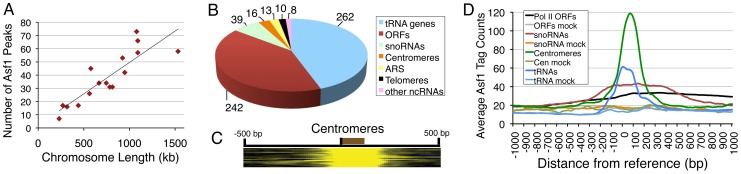
Chromosomal features enriched with Asf1. (A) Yeast chromosomes show similar density of Asf1 peaks. Total number of assigned Asf1-peaks for each chromosome when plotted against the chromosome length shows linear relationship. (B) Pie-chart showing different categories of genome-wide chromosomal features targeted by Asf1, number next to a category indicates total number of features occupied by Asf1 in that category. Out of 32, occupancy could be ascertained only on 10 telomeres. (C) Heat map shows Asf1 occupancy from blue (low) to yellow (high) at 500 bp upstream and downstream of the 5′ end (marked with a short bar) of 16 yeast centromeres. The brown bar marks the position of centromeres. (D) Comparison of average Asf1 occupancy on different genomic regions. Asf1 ChIP and mock sequencing tag counts on 1 kb region on both sides of a reference point on four genomic features were binned and the bin-wise average is plotted. Reference point is denoted by ‘0’ on the X-axis, which is TSS for tRNAs and pol II ORFs but SGD start co-ordinates for the others.

Since Asf1 is a general H3/H4 chaperone, we used peak calling criteria similar to nucleosome peak calling (peak size 150 bp and spacing of peaks by 300 bp), with a FDR of 0.1%. Analysis of the ChIP-sequencing data revealed only 590 chromosomal locations (Table S3) associated with Asf1 throughout the 16 chromosomes. The number of assigned Asf1 peaks on a chromosome shows a linear relationship with the chromosome length (Figure 1A), indicating that Asf1 is uniformly distributed on all chromosomes. Interestingly, ∼57% of all Asf1 targets are found in the inter-genic (between protein-coding genes) regions of diverse categories viz., transfer RNA (tRNA), small nuclear RNA (snRNA) and small nucleolar RNA (snoRNA) genes, autonomously replicating sequences (ARS) as well as centromeres (Figure 1B).

Asf1 occupies the gene body of almost all (262 out of 275, 95%) tRNA genes (Figure 1B) and other non-coding RNA genes (*SNR6*, *SNR52*, *RPR1* and *SCR1*) transcribed by pol III (Figure S2) as well as 242 ORFs (open reading frames) and more than 50% of the snoRNA genes, transcribed by pol II. Majority of these ORFs (Table S3) belong to the highly expressed category in the genome-wide gene expression data [42]. Using MIPS Functional Catalogue annotation to find whether these ORFs represent genes of a special functional category, we found that the genes for ribogenesis contribute the major fraction of the ORFs targeted by Asf1 (Table S4). Pol III targets, which are known sites of replication fork stalling [36], are highly transcribed. Even other highly transcribed genes and centromeres targeted by Asf1 are known sites of replication fork stalling [35], [43], where Asf1 is reported to help maintain replisome integrity [10], [11].

The presence of Asf1 on the telomeres agrees with its previously established role in telomeric silencing [44]. Since we used only unique reads to map Asf1 targets, peak finding program did not pick all telomeres (Figure 1B). In comparison, all the 16 centromeres in the budding yeast show a strong peak of Asf1 symmetrically centered at the middle of centromeric feature (Figure 1C). A comparison of the average occupancy of Asf1 on highly occupied groups of chromatin features revealed the highest levels on the centromeres followed by tRNAs while the lower Asf1 levels on the ORFs and snoRNA genes show a broad distribution along the length of gene (Figure 1D). As compared to *mcm* mutants, Asf1 has a minor role in chromosome maintenance [44], while no requirement of Asf1 for any centromeric function is reported in the budding yeast.

### Asf1 association at the pol II ORFs matches their transcription activity

**Figure 2:**
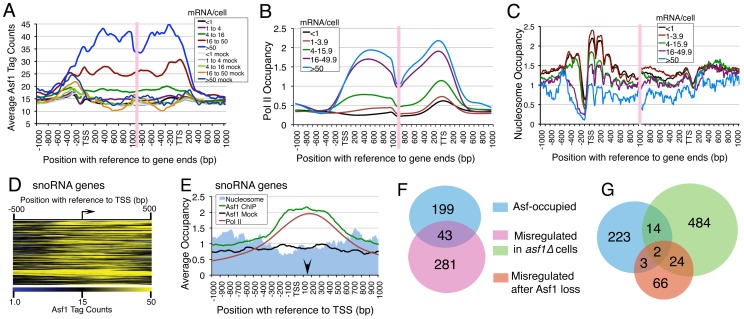
Asf1 association correlates with transcription activity of pol II. Average occupancy was calculated for (A) Asf1 ChIP and mock, (B) pol II, and (C) nucleosomes in the 2 kb region surrounding TSS or TTS of different classes of genes and plotted for the five categories based on the transcript abundance [42]. Legends box shows the color code, numbers indicate mRNA molecules/cell in the category. Vertical pink bar on the graphs represents the break in the middle. Panel A shows that the ChIP signals are well above the mock on highly transcribed genes. (D) Heat map depicting the Asf1 ChIP-Seq signal 500 bp upstream and downstream of the TSS (bent arrow) at snoRNA genes. Color gradient code is shown at the bottom. (E) Asf1 association at pol II-transcribed snoRNA genes, 1 kb upstream and 1 kb downstream of the TSS is compared with pol II and nucleosome occupancy profiles at the gene loci. Averages of Asf1 ChIP- and mock-Seq signals for 39 out of 77 genes are plotted. The vertical arrow denotes the gene 3′-end. (F) Venn intersections of Asf1-occupied (this study) genes with those misregulated in *asf1Δ* cells [28]. *P* value of the overlap is 3.9×10^−11^ (significant overlap). (G) Venn intersections of Asf1-occupied (this study) genes with those misregulated in *asf1Δ* cells [25] or after Asf1 depletion [25], with *P* values 0.118 and 0.42 respectively (insignificant overlaps).

Our Asf1 enrichment data have under-representation of ORFs (p value<2.2e-16). We divided ∼4000 pol-II transcribed ORFs into five categories, based on their transcriptional activity [42] and compared Asf1 occupancy with reported pol II profiles [33] on them. Pol II ORFs falling within 1 kb window of inter-genic (tRNA genes, snoRNAs genes, centromeres) and telomeric Asf1 targets were eliminated from this analysis to avoid contribution from these features. A heat map comparison of the occupancy data on 500 genes expressed at highest or lowest levels (Figure S3) shows that Asf1 association increases with an increase in transcriptional activity. We aligned the Asf1 occupancy data on every ORF in each category according to its transcription start site (TSS) or transcription termination site (TTS). Average occupancies in a window of 1 kb upstream and downstream of TSS or TTS on genes belonging to a category are plotted together in the Figure 2A. Asf1 occupancy shows higher levels at highly transcribed ORFs and vice versa (Figure 2A), indicating a positive correlation with the transcriptional status of a given ORF. Accordingly, we did not find Asf1 on *ARG1*, a gene reported as repressed under growth on enriched media [24]. A comparison of the Asf1 occupancy profile with the average pol II occupancy for the same set of five categories of ORFs (Figure 2B) revealed a high similarity between the two. Further, the peak heights of Asf1 and pol II at the 5′ and 3′ ends of genes match better for highly expressed genes (>16 mRNA copies/cell, Figures 2A and B). Transcription-dependent binding of Asf1 to the coding region of some inducible genes was shown earlier to facilitate pol II elongation [30]. Above results find that even on the constitutive pol II-transcribed ORFs, higher the Asf1 association, higher is the transcription activity.

### Asf1 and Nucleosome occupancies are inversely related on pol II-transcribed genes

Yeast pol II-transcribed genes generally have a nucleosome-free region (NFR) at the 5′ end of genes, flanked by two positioned nucleosomes, −1 and +1 [45]. Another NFR at 3′-end of genes coincides with the TTS of the genes. A comparison with the data on nucleosome occupancy data [46] revealed that relatively constant levels of Asf1 are found at the coding region while the position of the dip near TSS for ORFs expressed at moderate or low levels matches with the NFR in nucleosome profile (cf. Figures 2A and C). Importantly, a prominent peak of Asf1 is seen at the nucleosome preceding the TTS but not the downstream nucleosome on highly transcribed genes. Similarly, Asf1 occupancy towards the 5′ half of the genes shows negative correlation with nucleosome occupancy (cf. Figures 2A and C). Our data agree with previous findings that histones and pol II occupancies are inversely related and Asf1 assists pol II elongation by evicting histones from gene body and ends [30], [32]–[34]. Similar correlation of the Asf1, pol II and nucleosome occupancy is found on another pol II-transcribed gene class, the snoRNA genes. Asf1 shows a broad peak covering these genes (Figure 2D). Its distribution matches well with the pol II profile, while showing a negative correlation with nucleosome occupancy profile (Figure 2E).

### Asf1 binding is not required for its influence on the global gene expression

Global gene expression studies in the budding yeast have revealed transcriptional misregulation of many genes upon deletion of Asf1 [25], [28]. We compared the list of Asf1-occupied ORFs from our ChIP-Seq data with the list of the genes misregulated upon Asf1 deletion in both the reports (Figures 2F and G). In the two lists of misregulated genes in *asf1Δ* cells (color coded pink and green), only 89 genes were common, while only 26 genes were found misregulated (Figure 2G) under the two conditions of Asf1 absence; Asf1 deletion or conditional depletion [25]. In all, we found only a small number (∼15%) of misregulated genes occupied by Asf1. Asf1 occupies 43 out of 324 [28] and only 16 out of 524 [25] such genes (Figures 2F and 2G). Out of the 95 genes misregulated when Asf1 is depleted [25], only 5 show binding of Asf1 in this study (Figure 2G). Asf1-occupied genes (Table S5) and the misregulated genes in *asf1*Δ cells [25] do not show any functional overlap also. This analysis shows that Asf1 association with most of the genes misregulated in its absence is not stable. Accordingly, we did not find Asf1 on *HUG1* and *RNR3* loci, earlier reported to show an increase in the *asf1*ΔZ cells and a requirement of Asf1-dependent H3K56 acetylation for their transcriptional regulation [28].

### Asf1 deletion increases RNA levels from the pol III-transcribed genes

**Figure 3:**
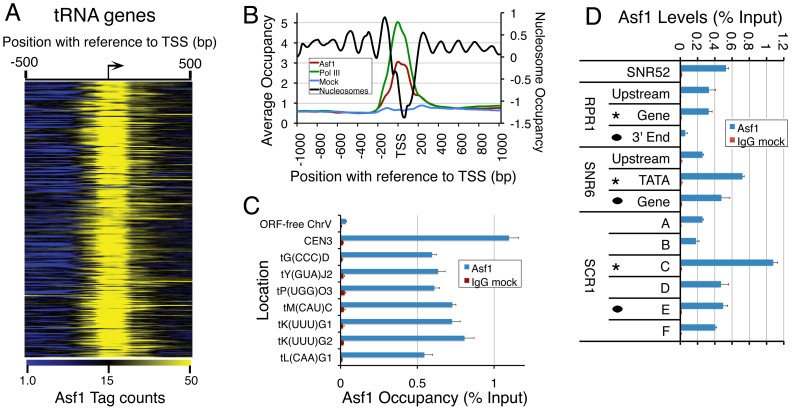
Asf1 association reduces pol III-transcribed gene product levels. (A) Heat map of Asf1 occupancy within 500 bp upstream and downstream of TSS (bent arrow) on all tRNA genes. Color gradient code is shown at the bottom. (B) Average Asf1 ChIP and mock occupancy values 1 kb upstream and 1 kb downstream of TSS of all tRNAs were divided by 20 and plotted on the same scale to compare with pol III and nucleosome occupancy profiles [41] at the tRNA gene loci. Average gene length is taken as 100 bp. (C) and (D) ChIP validations at various loci by Real Time PCR, using primers detailed in the Table S2. Occupancy on an ORF-free locus on chromosome V is shown as negative control. The values represent average of three independent experiments; error bars indicate standard deviation. In the panel D, * and dot mark the 5′- and 3′- ends respectively of the genes.

Other than centromeres, Asf1 occupancy on tRNA genes (268 out of 590, ∼50%) is highest of all targets (Figures 1C, 1D, 3A). Using previously published data [41], we compared average occupancy profile of Asf1, pol III and nucleosomes on 274 tRNA genes of yeast (Figure 3B). Unlike nucleosomes, Asf1 occupies the entire coding region of tRNA genes (Figure 3B). It shows a distribution nearly identical to that of pol III, with a peak centered at TSS and positive correlation coefficient 0.54 (R^2^ 0.29) between the two. Validation of the ChIP-Seq data for Asf1 occupancy at different pol III-transcribed genes by ChIP-qPCR shows high occupancy of Asf1 (Figures 3C,D) on tRNA genes and centromeric DNA, as compared to other ORF-free intergenic regions (Figure 3C). On non-tRNA genes, Asf1 levels at the 5′ ends are found higher than at the 3′ ends (Figure 3D).

Asf1 occupies the nucleosome-free bodies of the pol III-transcribed genes, which show transcribed regions are nucleosome-free while nucleosomes are found on both 5′- and 3′- flanking regions of the genes (Figure S4A, [41]). Its association with pol III-transcribed genes and an increase in levels of *SNR52* and some tRNAs in *asf1*Δ cells have been suggested as related to transcription activity [30] and RNA stability [28]. Using Real Time qPCR method, we found 1.5–2.5 fold increase in RNA levels from other tRNA (representing five different tRNA families) as well as non-tRNA (*SNR6* and *SNR52*) pol III-transcribed genes, in *asf1*Δ cells as compared to the wild-type cells, although *RPR1* shows only marginal increase (Figure S4B).

A defective nucleosome assembly but no change in the nucleosome density or the core histone levels in *asf1Δ* cells were reported earlier [14], [18]. The increase of RNA and a lower nucleosome occupancy on all (except *SNR6*) Asf1 targets (Figure 3) in *asf1Δ* cells (Figures S4C and D) in this study, imply that the higher RNA levels in *asf1Δ* cells could be related to the lower nucleosome occupancy on transcribed regions. Therefore, the relationship of Asf1 with transcription and nucleosome occupancy on pol III and pol II-transcribed genes may be different.

### Asf1 influences histone exchange at the 3′-end of pol III-transcribed genes

**Figure 4:**
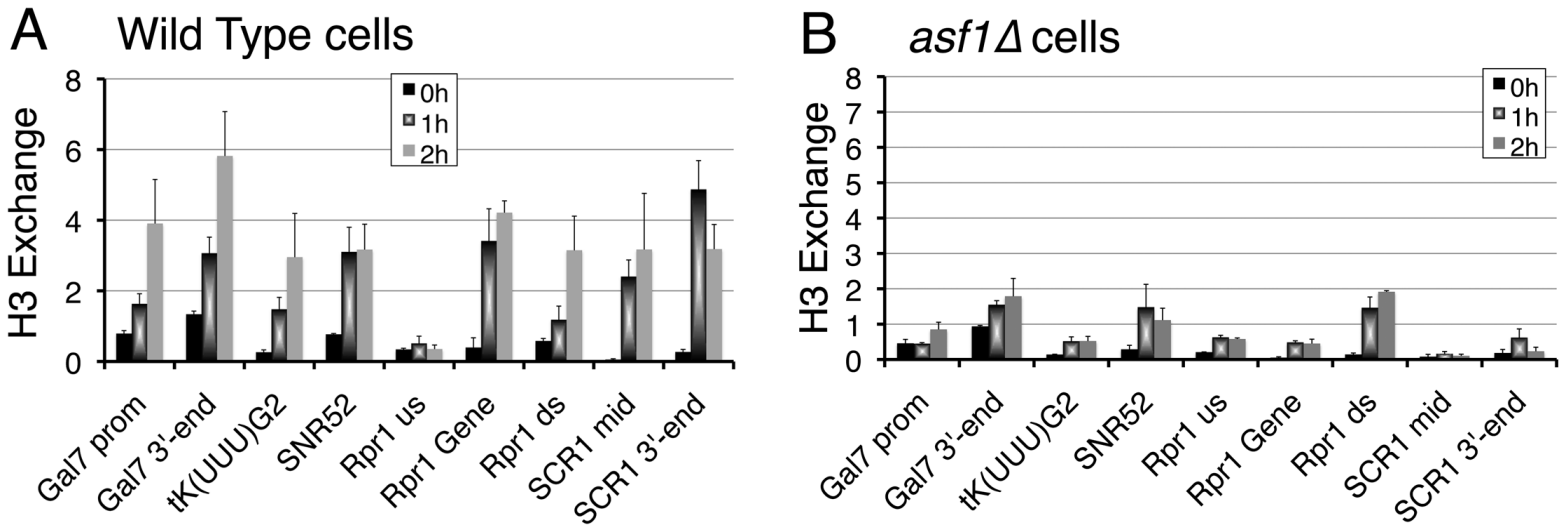
Replication-independent H3 exchange on pol III-transcribed genes. (A) and (B) Time-course analysis of histone H3 exchange at different loci in wild-type (A) versus *asf1Δ* (B) cells is shown. Histone exchange assay by ChIP-qPCR analysis was made to follow the H3 exchange [34] at various pol III-transcribed genes. Averages of ChIP data for three independent experiments with error bars are shown.

Asf1 has been implicated in genome-wide replication-independent H3 exchange at ∼2000 pol II ORFs, tRNA genes and snoRNAs genes, although the direct binding of Asf1 to these features has not been shown [33], [34]. A comparison of genome-wide exchange data [33] with our Asf1 occupancy data revealed that despite being occupied by Asf1, one-fourth of Asf1-occupied pol II genes and one-third of the tRNA genes do not show transcription-dependent H3/H4 exchange. Our measurements at some of these genes showed an Asf1-dependent H3/H4 exchange on the promoter and 3′ end of the positive control gene, *GAL7* (Figure 4), but not on any of the *SNR6* gene regions (Figure S4E). The level of H3/H4 exchange at the *RPR1* downstream region, where Asf1 and nucleosome occupancies are very low, shows a very small (∼1.6 fold) decrease in *asf1Δ* cells (cf. Figures 3D, S4C, 4A and B). Out of the six selected regions on *SCR1* (Figure S1B), high exchange rate is seen towards 3′end and on the gene body (Figure S4E). Similarly, high exchange rate on the gene bodies of *SNR52*, *RPR1* and two of tested tRNA genes could be seen (Figures 4A and S4E). In contrast, despite the presence of Asf1 at the 5′ end and upstream regions of *SCR1*, *RPR1* and whole of *SNR6* gene (Figure 3D), little exchange of H3/H4 is seen on these regions (Figures 4A and S4E). Thus, pol III transcription on these genes is accompanied by rapid histone exchange at the 3′ end and transcribed gene regions. This exchange is Asf1-dependent as it is abolished in *asf1Δ* cells (cf. Figures 4A and B) and Asf1 is found on all these locations (Figures 3C and D). Nevertheless, the correlation between Asf1 occupancy and histone exchange on different pol III-transcribed genes is not direct (cf. Figures 3C, 3D, 4A and S4E), similar to a lack of correlation between global H3 exchange [33] and our Asf1 occupancy data.

### Asf1 targets are co-occupied by the condensin complex

**Figure 5:**
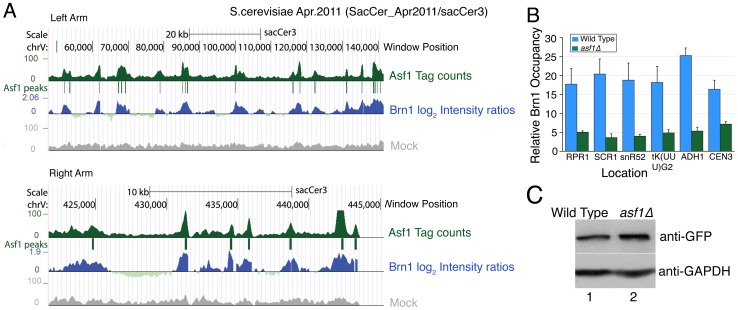
Asf1 facilitates loading of condensin on chromosomes. (A) Co-localization of Asf1 and condensin on yeast chromosome V. Genome-wide occupancy data of Asf1 (this study) and condensin subunit Brn1 [47] were compared. Screen shots of the distribution profiles of normalized occupancies of the condensin subunit Brn1 (blue peaks, log_2_ intensity ratios), Asf1 (green peaks, tag counts) and Asf1-mock (grey peaks, tag counts) along the length of chromosome V were visualized in UCSC genome browser. A close up view of ∼80 kb region on its left arm (upper panel) and ∼20 kb region on the right arm (lower panel) shows a highly similar distribution of Asf1 and Brn1 peaks. Exact positions of the assigned Asf1 peaks are marked. (B) ChIP analysis of Brn1 occupancy at different condensin-binding sites in wild-type and *asf1Δ* cells (C) Total cellular levels of Brn1-GFP protein in wild-type (lane 1) and *asf1Δ* (lane 2) cells are same. GAPDH was used as a loading control in this Western blot.

We noticed that Asf1 targets found in this study matched with the reported genome-wide targets of condensin [47] including tRNA, snoRNA and ribosomal protein encoding genes. The linear distribution of assigned Asf1 peaks along the length of all chromosomes (Figure 1A) is also similar to that for condensin in the earlier report [47]. Therefore, we calculated the normalized occupancy [47] of Brn1, a condensin subunit, at the assigned Asf1 peaks and found that the majority of Asf1 targets (493 out of 607, ∼81%), are co-occupied by Brn1 with a positive correlation coefficient 0.52 (R^2^ = 0.27). A comparison of the peak distributions and a close up view of both the arms of chromosome V show that Asf1 and Brn1 peak distributions are nearly identical (Figure 5A, cf. two panels). ChIP-qPCR measurements show reduced Brn1 levels on some of its tested target sites in *asf1Δ* cells (Figure 5B) although Brn1 levels do not change in the deletion mutant (Figure 5C).

Similar to condensin mutants, *asf1Δ* yeast cells, though viable, accumulate in G2/M phase of cell cycle [13], [17]. In the context of the known functions of both in the chromatin structure and higher order chromatin organization, the highly similar Asf1 and Brn1 occupancy profiles along with decreased Brn1 occupancy upon Asf1 deletion imply a facilitatory role of Asf1 in condensin loading on the chromosomes.

## Discussion

This study brings forth a functional overlap between transcription, replication, and chromosome architecture mediated by Asf1. Asf1 binding to chromatin has been envisaged as non-specific [24], [28]. Its binding to only a fraction of the pol II-transcribed genes suggests that its reported roles in genome-wide histone exchange and transcription regulation may be indirect or redundant with other chaperones. However, the absence of physical association with most of the functional targets may not be unusual. It has also been observed in earlier genome-wide studies on some other proteins involved in regulating chromatin structure and dynamics, suggesting a transient association with chromatin [48]–[51].

Highly transcribed regions are reported “hyper-ChIPable” where several unrelated and biologically non-meaningful proteins also show enrichment [52]. However, literature has ample research evidence for functional relevance of Asf1 binding to highly transcribed loci [30], [32], [33]. Accordingly, high association of Asf1 with such regions, found in our ChIP-seq data may not be artefactual. Asf1-associated genomic regions include both highly transcribed features and heterochromatic regions. Asf1 role in maintaining replisome integrity [11] suggests an increased dwelling time at highly transcribed genes, which are also reported to be the replication fork pause sites. This comparatively non-transient association at certain sites may also be the reason that higher Asf1 levels are found at replication fork pause sites like tRNA genes (highly transcribed) and centromeres (non-transcribed).

It was earlier demonstrated that high histone exchange seen at NFRs represents a continuous process that disrupts nucleosomes and maintains DNA accessibility [53]. This may be the reason that NFR on tRNA genes is actively maintained by participation of several ATP-dependent chromatin remodelers [41]. The Asf1-dependent histone exchange at the 3′ end of the pol III-transcribed genes may also be related to the higher nucleosome dynamics at the downstream end for these genes, reported earlier [41], probably to keep the gene terminator accessible. However, further experiments are required to understand how Asf1 regulates pol III transcription.

Asf1 is required for H3K56 acetylation by Rtt109 [20] and in its absence, eviction of unacetylated nucleosomes is reduced [54]. On the pol II-transcribed *PHO5* gene, higher H3 levels were found in *asf1Δ* cells [37] and similar to *HUG1* and *RNR3* loci [28], its activation required H3K56 acetylation [54], again suggesting that Asf1 regulates genes due to its requirement for H3K56 acetylation, which in turn, promotes nucleosome eviction by Asf1. A strong correlation has been reported between H3 exchange and H3K56 acetylation, genome-wide (r = 0.865) as well as on tRNA genes (correlation coefficient 0.844; R^2^ = 0.712) in the same data [33]. Therefore, role of Asf1 in histone-exchange may be facilitatory, locus-specific and indirect. In agreement, a recent study has computed that the histone H3 exchange pattern in yeast is specific for each gene and does not depend on transcription rate of the gene [55].

Further probing of the functional and physiological implications of its high association with centromeres and genome-wide co-localization with condensin may reveal novel functions of Asf1. An earlier study [47] had reported a partial correlation of condensin occupancy with its other possible loaders on the chromatin. In this study, loss of Brn1 from its binding sites in Asf1 deletion cells suggests a requirement for Asf1 in condensin loading. A physical association between the two is a possibility, which has not been tested or reported yet. Cohesin loader Scc2/4 is also required for loading condensin but does not interact with it [47] while transcription factor of RNA polymerase III, TFIIIC and condensin co-localize to tRNA genes but not to centromeres [47]. It was also found in the same study that though TFIIIC binding sites may be the minimum condensin-binding regions, deletion of tRNA gene sequence does not abolish condensin binding [47], suggesting involvement of other facilitatory factors for condensin loading. In light of these observations, Asf1 could be this missing factor, as a dramatic fall in Brn1 occupancy is seen on all tested loci in *asf1Δ* cells. Since Asf1 is a highly conserved H3/H4 chaperone, its targets in higher eukaryotes may be similar. The outcomes of this study also propose that identifying genome-wide targets of other histone chaperones can resolve known redundancy and specific or novel functions of different histone chaperones.

## Supporting Information

Figure S1 **ChIP efficiency check.** (A) Quality check for ChIP DNA preparations. After crosslinking, chromatin was fragmented using Bioruptor sonicator to a mean size of 150–300 bp. Chromatin fragments were separated and an aliquot was analyzed by agarose gel electrophoresis. Input DNA preparations for both replicates are shown (lane 1 and 2); lane M depicts 100 bp-ladder, used as size marker. ChIP and Mock experiments were performed using the same input DNA (input 1 for replicate 1 and mock, input 2 for replicate 2). Schematic diagrams in the right panels show amplicon positions used for ChIP-qPCR experiments on *SCR1* (B), *RPR1* (C) and *SNR6* (D) loci. Two genes transcribed by RNA polymerase III, viz. *SCR1* and *RPR1*, were selected due to their relatively longer length than other pol III-transcribed genes.
(PDF)

Figure S2 **Asf1 association profile at some of the pol III-transcribed genes.** Screen shots from UCSC genome browser showing Asf1 association at (A) *SNR52*, (B) *SNR6*, (C) *SCR1* and (D) *RPR1* genes. Y-axis represents normalized Asf1 tag counts; mock tag count shows background. Distribution of Asf1 (enriched Asf1 peaks in green; background in blue) is shown at different pol III-transcribed genes.
(PDF)

Figure S3 **Asf1 preferentially occupies the genes highly transcribed by pol II.** Heat maps depicting Asf1 ChIP-Seq signal at different pol II ORFs. Color code is shown at the bottom. Bent arrow represents TSS. Asf1 occupancy 500 bp upstream and downstream of the TSS for all pol II ORFs was calculated. Heat maps of 500 lowest expressed (A, bottom 500) and 500 highly expressed (B, top 500) ORFs are shown. Vertical arrow shows increasing order of transcription activity of ORFs in each category.
(PDF)

Figure S4 **Asf1 deposits nucleosomes on pol III-transcribed genes.** (A) Data from the previous study [41] were used to generate the heat map of nucleosome occupancy 1 kb upstream and 1 kb downstream of the TSS (bent, green arrow) of the four non-tRNA pol III-transcribed genes. Genes reside in the NFR on the right side of their TSS. Gene body is denoted by grey bars. Color code for the occupancy gradient is shown at the bottom of the panel. (B) RNA levels of some of the pol-III transcribed genes in the *asf1*Δ cells, as compared to RNA levels in the *wild*-type cells, set to 1. SCR1 was used as an internal control. The averages of five independent experiments with error bars are shown. (C) and (D) show results of nucleosome occupancy measured on various pol III-transcribed genes in wild-type and *asf1*Δ cells. Mononucleosomal DNA isolated from chromatin digested with micrococcal nuclease *in situ* was used for qPCR. Averages from three independent estimations with error bars are shown. Amplicons A–F for SCR1 regions (panel D) are described in the Figure S1. (E) Replication-independent histone H3 exchange occurs in the coding region of Pol III-transcribed genes. Time-course analysis of replication-independent H3 exchange [34] on some of the pol III-transcribed genes is shown. ChIP-qPCR analysis was used to follow the H3 exchange at various genes.
(PDF)

Table S1 List of the yeast strains used.
(PDF)

Table S2 List of the Primers used.
(PDF)

Table S3 Different categories of the genome-wide chromosomal features targeted by Asf1.
(PDF)

Table S4 Functional distribution of Asf1-occupied 242 pol II genes.
(PDF)

Table S5 Transcription activity at 242 ASf1-occupied pol II ORFs.
(XLSX)

Methods S1 This file contains details of the methods followed and the supplementary references cited in the Supporting Information files.
(PDF)

## References

[1] Misteli T (2013) The cell biology of genomes: bringing the double helix to life. Cell 152: 1209–1212. 23498929 10.1016/j.cell.2013.02.048 PMC6318791

[2] Henikoff S (2008) Nucleosome destabilization in the epigenetic regulation of gene expression. Nat Rev Genet 9: 15–26. 18059368 10.1038/nrg2206

[3] Bhargava P (2010) Epigenetics to proteomics: from yeast to brain. Proteomics 10: 749–770. 19834912 10.1002/pmic.200900464

[4] Tyler JK (2002) Chromatin assembly. Cooperation between histone chaperones and ATP-dependent nucleosome remodeling machines. Eur J Biochem 269: 2268–2274. 11985607 10.1046/j.1432-1033.2002.02890.x

[5] De Koning L, Corpet A, Haber JE, Almouzni G (2007) Histone chaperones: an escort network regulating histone traffic. Nat Struct Mol Biol 14: 997–1007. 17984962 10.1038/nsmb1318

[6] Park YJ, Luger K (2008) Histone chaperones in nucleosome eviction and histone exchange. Curr Opin Struct Biol 18: 282–289. 18534842 10.1016/j.sbi.2008.04.003 PMC2525571

[7] Ransom M, Dennehey BK, Tyler JK (2010) Chaperoning histones during DNA replication and repair. Cell 140: 183–195. 20141833 10.1016/j.cell.2010.01.004 PMC3433953

[8] Mousson F, Ochsenbein F, Mann C (2007) The histone chaperone Asf1 at the crossroads of chromatin and DNA checkpoint pathways. Chromosoma 116: 79–93. 17180700 10.1007/s00412-006-0087-z

[9] Zunder RM, Rine J (2012) Direct interplay among histones, histone chaperones, and a chromatin boundary protein in the control of histone gene expression. Mol Cell Biol 32: 4337–4349. 22907759 10.1128/MCB.00871-12 PMC3486138

[10] Schulz LL, Tyler JK (2006) The histone chaperone ASF1 localizes to active DNA replication forks to mediate efficient DNA replication. FASEB J 20: 488–490. 16396992 10.1096/fj.05-5020fje

[11] Franco AA, Lam WM, Burgers PM, Kaufman PD (2005) Histone deposition protein Asf1 maintains DNA replisome integrity and interacts with replication factor C. Genes Dev 19: 1365–1375. 15901673 10.1101/gad.1305005 PMC1142559

[12] Groth A, Corpet A, Cook AJ, Roche D, Bartek J, et al (2007) Regulation of replication fork progression through histone supply and demand. Science 318: 1928–1931. 18096807 10.1126/science.1148992

[13] Tyler JK, Adams CR, Chen SR, Kobayashi R, Kamakaka RT, et al (1999) The RCAF complex mediates chromatin assembly during DNA replication and repair. Nature 402: 555–560. 10591219 10.1038/990147

[14] Adkins MW, Tyler JK (2004) The histone chaperone Asf1p mediates global chromatin disassembly in vivo. J Biol Chem 279: 52069–52074. 15452122 10.1074/jbc.M406113200

[15] Green EM, Antczak AJ, Bailey AO, Franco AA, Wu KJ, et al (2005) Replication-independent histone deposition by the HIR complex and Asf1. Curr Biol 15: 2044–2049. 16303565 10.1016/j.cub.2005.10.053 PMC2819815

[16] Takahata S, Yu Y, Stillman DJ (2009) FACT and Asf1 regulate nucleosome dynamics and coactivator binding at the HO promoter. Mol Cell 34: 405–415. 19481521 10.1016/j.molcel.2009.04.010 PMC2767235

[17] Ramey CJ, Howar S, Adkins M, Linger J, Spicer J, et al (2004) Activation of the DNA damage checkpoint in yeast lacking the histone chaperone anti-silencing function 1. Mol Cell Biol 24: 10313–10327. 15542840 10.1128/MCB.24.23.10313-10327.2004 PMC529054

[18] Prado F, Cortes-Ledesma F, Aguilera A (2004) The absence of the yeast chromatin assembly factor Asf1 increases genomic instability and sister chromatid exchange. EMBO Rep 5: 497–502. 15071494 10.1038/sj.embor.7400128 PMC1299049

[19] Adkins MW, Carson JJ, English CM, Ramey CJ, Tyler JK (2007) The histone chaperone anti-silencing function 1 stimulates the acetylation of newly synthesized histone H3 in S-phase. J Biol Chem 282: 1334–1340. 17107956 10.1074/jbc.M608025200

[20] Recht J, Tsubota T, Tanny JC, Diaz RL, Berger JM, et al (2006) Histone chaperone Asf1 is required for histone H3 lysine 56 acetylation, a modification associated with S phase in mitosis and meiosis. Proc Natl Acad Sci U S A 103: 6988–6993. 16627621 10.1073/pnas.0601676103 PMC1459006

[21] Driscoll R, Hudson A, Jackson SP (2007) Yeast Rtt109 promotes genome stability by acetylating histone H3 on lysine 56. Science 315: 649–652. 17272722 10.1126/science.1135862 PMC3334813

[22] Han J, Zhou H, Li Z, Xu RM, Zhang Z (2007) Acetylation of lysine 56 of histone H3 catalyzed by RTT109 and regulated by ASF1 is required for replisome integrity. J Biol Chem 282: 28587–28596. 17690098 10.1074/jbc.M702496200

[23] Chen CC, Carson JJ, Feser J, Tamburini B, Zabaronick S, et al (2008) Acetylated lysine 56 on histone H3 drives chromatin assembly after repair and signals for the completion of repair. Cell 134: 231–243. 18662539 10.1016/j.cell.2008.06.035 PMC2610811

[24] Lin LJ, Schultz MC (2011) Promoter regulation by distinct mechanisms of functional interplay between lysine acetylase Rtt109 and histone chaperone Asf1. Proc Natl Acad Sci U S A 108: 19599–19604. 22106264 10.1073/pnas.1111501108 PMC3241807

[25] Zabaronick SR, Tyler JK (2005) The histone chaperone anti-silencing function 1 is a global regulator of transcription independent of passage through S phase. Mol Cell Biol 25: 652–660. 15632066 10.1128/MCB.25.2.652-660.2005 PMC543432

[26] Korber P, Barbaric S, Luckenbach T, Schmid A, Schermer UJ, et al (2006) The histone chaperone Asf1 increases the rate of histone eviction at the yeast PHO5 and PHO8 promoters. J Biol Chem 281: 5539–5545. 16407267 10.1074/jbc.M513340200

[27] Adkins MW, Williams SK, Linger J, Tyler JK (2007) Chromatin disassembly from the PHO5 promoter is essential for the recruitment of the general transcription machinery and coactivators. Mol Cell Biol 27: 6372–6382. 17620413 10.1128/MCB.00981-07 PMC2099613

[28] Minard LV, Williams JS, Walker AC, Schultz MC (2011) Transcriptional regulation by Asf1: new mechanistic insights from studies of the DNA damage response to replication stress. J Biol Chem 286: 7082–7092. 21190944 10.1074/jbc.M110.193813 PMC3044965

[29] Schwabish MA, Struhl K (2004) Evidence for eviction and rapid deposition of histones upon transcriptional elongation by RNA polymerase II. Mol Cell Biol 24: 10111–10117. 15542822 10.1128/MCB.24.23.10111-10117.2004 PMC529037

[30] Schwabish MA, Struhl K (2006) Asf1 mediates histone eviction and deposition during elongation by RNA polymerase II. Mol Cell 22: 415–422. 16678113 10.1016/j.molcel.2006.03.014

[31] Thiriet C, Hayes JJ (2005) Replication-independent core histone dynamics at transcriptionally active loci in vivo. Genes Dev 19: 677–682. 15769942 10.1101/gad.1265205 PMC1065721

[32] D]ion MF, Kaplan T, Kim M, Buratowski S, Friedman N, et al (2007) Dynamics of replication-independent histone turnover in budding yeast. Science 315: 1405–1408. 17347438 10.1126/science.1134053

[33] Rufiange A, Jacques PE, Bhat W, Robert F, Nourani A (2007) Genome-wide replication-independent histone H3 exchange occurs predominantly at promoters and implicates H3 K56 acetylation and Asf1. Mol Cell 27: 393–405. 17679090 10.1016/j.molcel.2007.07.011

[34] Jamai A, Imoberdorf RM, Strubin M (2007) Continuous histone H2B and transcription-dependent histone H3 exchange in yeast cells outside of replication. Mol Cell 25: 345–355. 17289583 10.1016/j.molcel.2007.01.019

[35] Azvolinsky A, Giresi PG, Lieb JD, Zakian VA (2009) Highly transcribed RNA polymerase II genes are impediments to replication fork progression in Saccharomyces cerevisiae. Mol Cell 34: 722–734. 19560424 10.1016/j.molcel.2009.05.022 PMC2728070

[36] Deshpande AM, Newlon CS (1996) DNA replication fork pause sites dependent on transcription. Science 272: 1030–1033. 8638128 10.1126/science.272.5264.1030

[37] Adkins MW, Howar SR, Tyler JK (2004) Chromatin disassembly mediated by the histone chaperone Asf1 is essential for transcriptional activation of the yeast PHO5 and PHO8 genes. Mol Cell 14: 657–666. 15175160 10.1016/j.molcel.2004.05.016

[38] Liu CL, Kaplan T, Kim M, Buratowski S, Schreiber SL, et al (2005) Single-nucleosome mapping of histone modifications in S. cerevisiae. PLoS Biol 3: e328. 16122352 10.1371/journal.pbio.0030328 PMC1195719

[39] Arimbasseri AG, Bhargava P (2008) Chromatin structure and expression of a gene transcribed by RNA polymerase III are independent of H2A.Z deposition. Mol Cell Biol 28: 2598–2607. 18268003 10.1128/MCB.01953-07 PMC2293117

[40] Mahapatra S, Dewari PS, Bhardwaj A, Bhargava P (2011) Yeast H2A.Z, FACT complex and RSC regulate transcription of tRNA gene through differential dynamics of flanking nucleosomes. Nucleic Acids Res 39: 4023–4034. 21266479 10.1093/nar/gkq1286 PMC3105386

[41] Kumar Y, Bhargava P (2013) A unique nucleosome arrangement, maintained actively by chromatin remodelers facilitates transcription of yeast tRNA genes. BMC Genomics 14: 402. 23767421 10.1186/1471-2164-14-402 PMC3698015

[42] Holstege FC, Jennings EG, Wyrick JJ, Lee TI, Hengartner CJ, et al (1998) Dissecting the regulatory circuitry of a eukaryotic genome. Cell 95: 717–728. 9845373 10.1016/s0092-8674(00)81641-4

[43] Greenfeder SA, Newlon CS (1992) Replication forks pause at yeast centromeres. Mol Cell Biol 12: 4056–4066. 1508202 10.1128/mcb.12.9.4056-4066.1992 PMC360298

[44] Le S, Davis C, Konopka JB, Sternglanz R (1997) Two new S-phase-specific genes from Saccharomyces cerevisiae. Yeast 13: 1029–1042. 9290207 10.1002/(SICI)1097-0061(19970915)13:11<1029::AID-YEA160>3.0.CO;2-1

[45] Jansen A, Verstrepen KJ (2011) Nucleosome positioning in Saccharomyces cerevisiae. Microbiol Mol Biol Rev 75: 301–320. 21646431 10.1128/MMBR.00046-10 PMC3122627

[46] Brogaard K, Xi L, Wang JP, Widom J (2012) A map of nucleosome positions in yeast at base-pair resolution. Nature 486: 496–501. 22722846 10.1038/nature11142 PMC3786739

[47] D'Ambrosio C, Schmidt CK, Katou Y, Kelly G, Itoh T, et al (2008) Identification of cis-acting sites for condensin loading onto budding yeast chromosomes. Genes Dev 22: 2215–2227. 18708580 10.1101/gad.1675708 PMC2518811

[48] Ng HH, Robert F, Young RA, Struhl K (2002) Genome-wide location and regulated recruitment of the RSC nucleosome-remodeling complex. Genes Dev 16: 806–819. 11937489 10.1101/gad.978902 PMC186327

[49] Damelin M, Simon I, Moy TI, Wilson B, Komili S, et al (2002) The genome-wide localization of Rsc9, a component of the RSC chromatin-remodeling complex, changes in response to stress. Mol Cell 9: 563–573. 11931764 10.1016/s1097-2765(02)00475-6

[50] Whitehouse I, Rando OJ, Delrow J, Tsukiyama T (2007) Chromatin remodelling at promoters suppresses antisense transcription. Nature 450: 1031–1035. 18075583 10.1038/nature06391

[51] Sala A, Toto M, Pinello L, Gabriele A, Di Benedetto V, et al (2011) Genome-wide characterization of chromatin binding and nucleosome spacing activity of the nucleosome remodelling ATPase ISWI. EMBO J 30: 1766–1777. 21448136 10.1038/emboj.2011.98 PMC3102003

[52] Teytelman L, Thurtle DM, Rine J, van Oudenaarden A (2013) Highly expressed loci are vulnerable to misleading ChIP localization of multiple unrelated proteins. Proc Natl Acad Sci U S A 110: 18602–18607. 24173036 10.1073/pnas.1316064110 PMC3831989

[53] Mito Y, Henikoff JG, Henikoff S (2007) Histone replacement marks the boundaries of cis-regulatory domains. Science 315: 1408–1411. 17347439 10.1126/science.1134004

[54] Williams SK, Truong D, Tyler JK (2008) Acetylation in the globular core of histone H3 on lysine-56 promotes chromatin disassembly during transcriptional activation. Proc Natl Acad Sci USA 105: 9000–9005. 18577595 10.1073/pnas.0800057105 PMC2449354

[55] G-Viks I, Vingron M (2009) Evidence for gene-specific rather than transcription rate-dependent histone H3 exchange in yeast coding regions. PLoS Comput Biol 5: e1000282. 19197343 10.1371/journal.pcbi.1000282 PMC2625437

