# Supplementary figures and images for: Genome-Wide Mapping of Yeast Histone Chaperone Anti-Silencing Function 1 Reveals Its Role in Condensin Binding with Chromatin
Source: PLoS One. 2014 Sep 29;9(9):e108652. doi: 10.1371/journal.pone.0108652 (PMC4181348; doi:10.1371/journal.pone.0108652)

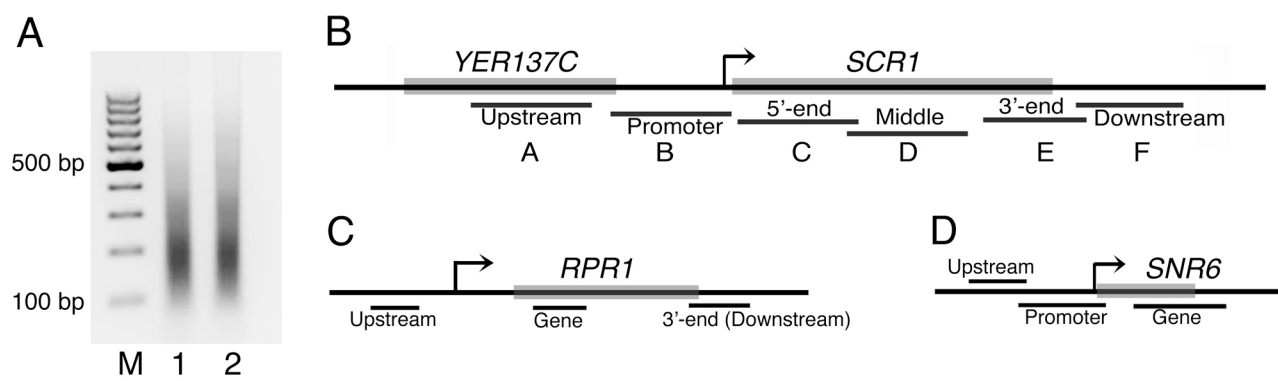

Figure S1

Supplement: Figure S1 — ChIP efficiency check. (A) Quality check for ChIP DNA preparations. After crosslinking, chromatin was fragmented using Bioruptor sonicator to a mean size of 150–300 bp. Chromatin fragments were separated and an aliquot was analyzed by agarose gel electrophoresis. Input DNA preparations for both replicates are shown (lane 1 and 2); lane M depicts 100 bp-ladder, used as size marker. ChIP and Mock experiments were performed using the same input DNA (input 1 for replicate 1 and mock, input 2 for replicate 2). Schematic diagrams in the right panels show amplicon positions used for ChIP-qPCR experiments on SCR1 (B), RPR1 (C) and SNR6 (D) loci. Two genes transcribed by RNA polymerase III, viz. SCR1 and RPR1, were selected due to their relatively longer length than other pol III-transcribed genes. (PDF) [file pone.0108652.s001.pdf]

Figure S2

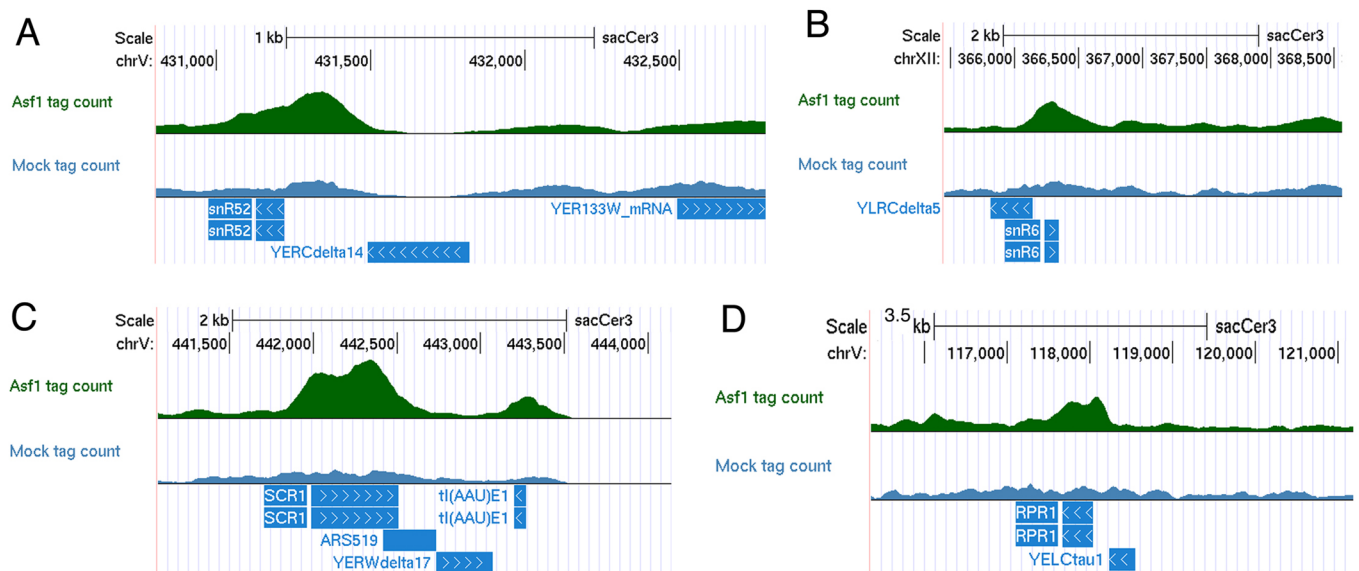

Supplement: Figure S2 — Asf1 association profile at some of the pol III-transcribed genes. Screen shots from UCSC genome browser showing Asf1 association at (A) SNR52, (B) SNR6, (C) SCR1 and (D) RPR1 genes. Y-axis represents normalized Asf1 tag counts; mock tag count shows background. Distribution of Asf1 (enriched Asf1 peaks in green; background in blue) is shown at different pol III-transcribed genes. (PDF) [file pone.0108652.s002.pdf]

Figure S3

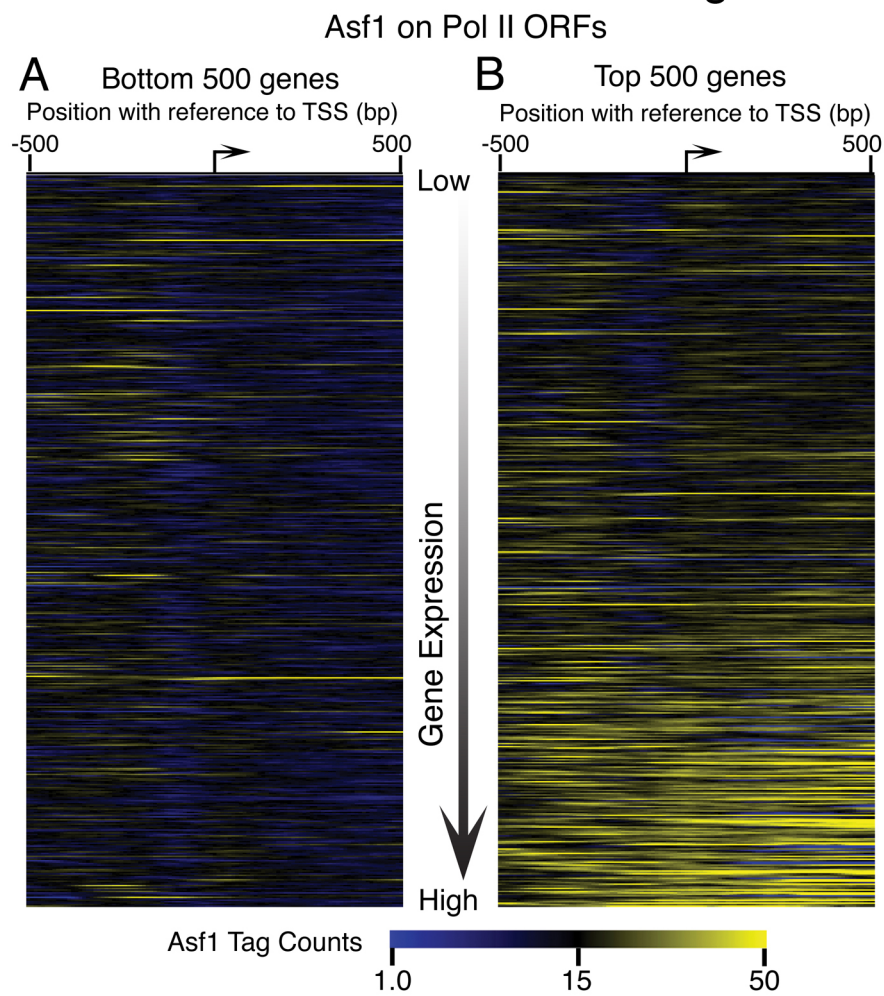

Supplement: Figure S3 — Asf1 preferentially occupies the genes highly transcribed by pol II. Heat maps depicting Asf1 ChIP-Seq signal at different pol II ORFs. Color code is shown at the bottom. Bent arrow represents TSS. Asf1 occupancy 500 bp upstream and downstream of the TSS for all pol II ORFs was calculated. Heat maps of 500 lowest expressed (A, bottom 500) and 500 highly expressed (B, top 500) ORFs are shown. Vertical arrow shows increasing order of transcription activity of ORFs in each category. (PDF) [file pone.0108652.s003.pdf]

Figure S4

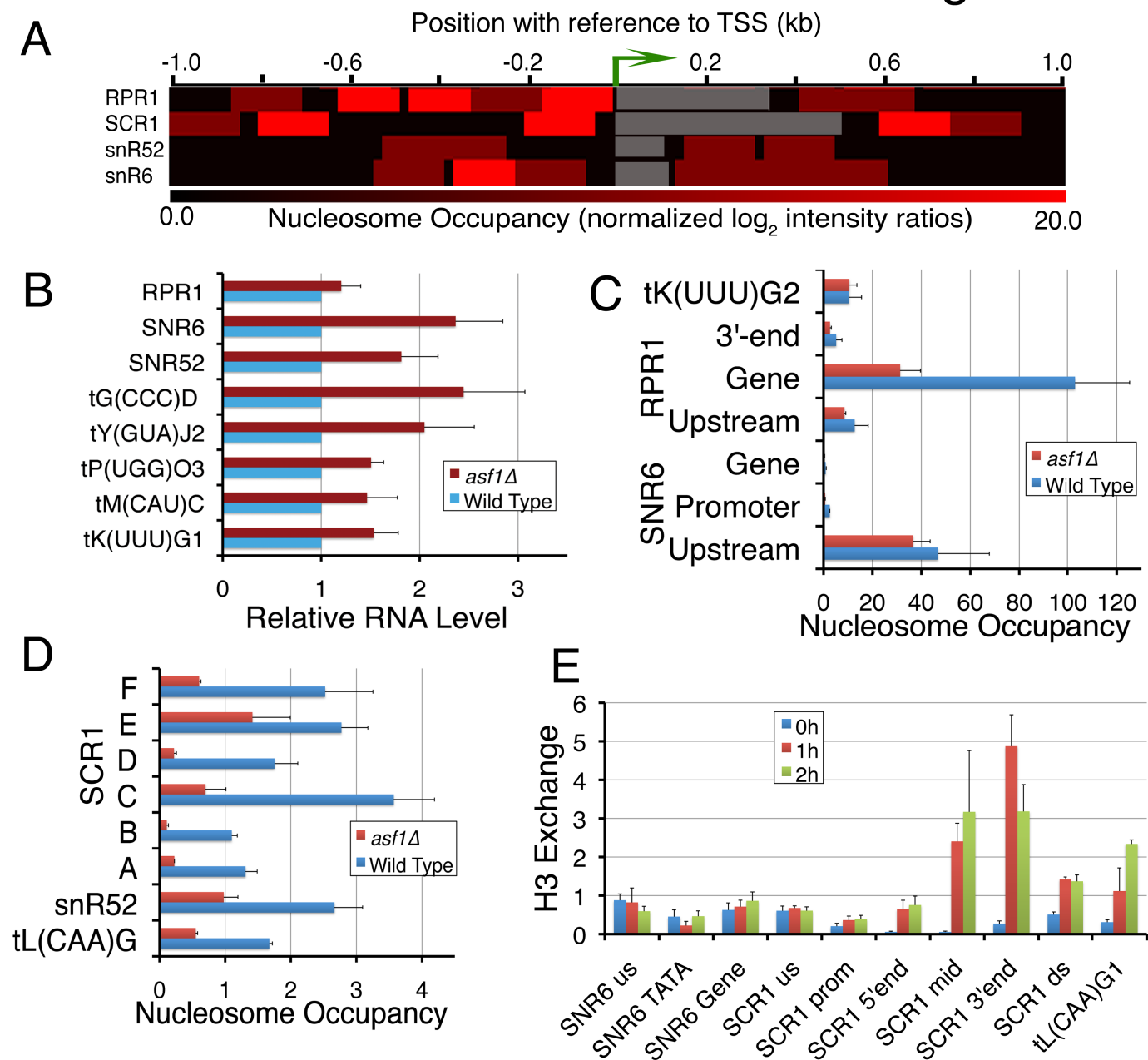

Supplement: Figure S4 — Asf1 deposits nucleosomes on pol III-transcribed genes. (A) Data from the previous study [41] were used to generate the heat map of nucleosome occupancy 1 kb upstream and 1 kb downstream of the TSS (bent, green arrow) of the four non-tRNA pol III-transcribed genes. Genes reside in the NFR on the right side of their TSS. Gene body is denoted by grey bars. Color code for the occupancy gradient is shown at the bottom of the panel. (B) RNA levels of some of the pol-III transcribed genes in the asf1Δ cells, as compared to RNA levels in the wild-type cells, set to 1. SCR1 was used as an internal control. The averages of five independent experiments with error bars are shown. (C) and (D) show results of nucleosome occupancy measured on various pol III-transcribed genes in wild-type and asf1Δ cells. Mononucleosomal DNA isolated from chromatin digested with micrococcal nuclease in situ was used for qPCR. Averages from three independent estimations with error bars are shown. Amplicons A–F for SCR1 regions (panel D) are described in the Figure S1. (E) Replication-independent histone H3 exchange occurs in the coding region of Pol III-transcribed genes. Time-course analysis of replication-independent H3 exchange [34] on some of the pol III-transcribed genes is shown. ChIP-qPCR analysis was used to follow the H3 exchange at various genes. (PDF) [file pone.0108652.s004.pdf]
