# Supplementary material for: Genome-Wide Mapping of Yeast Histone Chaperone Anti-Silencing Function 1 Reveals Its Role in Condensin Binding with Chromatin
Source: PLoS One. 2014 Sep 29;9(9):e108652. doi: 10.1371/journal.pone.0108652 (PMC4181348; doi:10.1371/journal.pone.0108652)
Supplement: Table S1 — List of the yeast strains used. (PDF) [file pone.0108652.s005.pdf]

Table S1: List of the yeast strains used in this study

Strain	Genotype	Parent strain	Source
YYK9	MATa ade2-1 can1-100 his3-11,15 leu2-3,112 trp1-1 ura3-1 bar1	W303-1A	[60]
yPB21	MATa ade2-1 can1-100 his3-11,15 leu2-3,112 trp1-1 ura3-1 bar1 asf1::KanMX	YYK9	This study
Asf1-18Myc	MATa ura3-52 trp1-Δ63 his3-Δ200 leu2::PET56 HIS3locus:int pJD30 -10IME2-Sc4251, LYS2 locus:int pJD52 X-Sc4251/LYS2 Asf1-18Myc:TRP1	JDY51	[30]
yPB32	MATa hta1-htb1Δ::LEU2, hta2-htb2Δ::TRP1, leu2-Δ1, ura3-52, trp1-Δ63, his3-Δ200/ pJH23 (FB1251) HIS3 CEN ARS HTA1, FLAG-HTB2 RPC160-9Myc:hph ASF1-6HA:KanMx	FY406	This study
FY406	MATa hta1-htb1Δ::LEU2, hta2-htb2Δ::TRP1, leu2-Δ1, ura3-52, trp1-Δ63, his3-Δ200/ pJH23 (FB1251) HIS3 CEN ARS HTA1, FLAG-HTB2		[62]
MSY104	MATa hta1-htb1Δ::LEU2 hta2-htb2::TRP1 leu2-Δ1 ura3-52 trp1-Δ63 his3-Δ200 asf1::KanMX pJH23(FB1251) HIS3 AMP CEN HTA1 FLAG-HTB2	FY406	[30]
GA2663	MAT a ade2-1 trp1-1 his3-11,15 ura3-1 leu2-3, 112 can1-100 brn1::BRN1-GFP-KanMX6	GA180	[61]
yPB41	MAT a ade2-1 trp1-1 his3-11,15 ura3-1 leu2-3, 112 can1-100 brn1::BRN1-GFP-KanMX6 asf1::hph	GA2663	This study
