# Supplementary material for: Genome-Wide Mapping of Yeast Histone Chaperone Anti-Silencing Function 1 Reveals Its Role in Condensin Binding with Chromatin
Source: PLoS One. 2014 Sep 29;9(9):e108652. doi: 10.1371/journal.pone.0108652 (PMC4181348; doi:10.1371/journal.pone.0108652)
Supplement: Table S2 — List of the Primers used. (PDF) [file pone.0108652.s006.pdf]

Table S2: List of the Primers used in this study

Gene/ Amplicon	Position reference	Forward primer	Reverse primer	Used for
TelVIR	chrVI: 269500 to 269637	GCGTAACAAAGCCATAATGCCTCC	CTCGTTAGGATCACGTTCGAATCC	ChIP
CEN3	chrIII: 114383 to 114508	AAGTCACATGATGATATTTGAT	ATTTCTTTTTTAACTTTCGGAA	ChIP
ORF-free chrV	chrV: 9716 to 9863	GGCTGTCAGAATATGGGGCCGTAGTA	CACCCCGAAGCTGCTTTCACAATAC	ChIP
COX3	+11 to +145	TAGAAAGAAGTAGACATCAACAA	TCATATTACCAATATAACCATGC	ChIP
GAL7 Promoter	-184 to -70	CTGTTGACCGTGATCCGAAGG	CCGACCTGCTTTTATATCTTTGCTA	ChIP
GAL7 3'-end	+898 to +998	CCTTTGAATGCGACTGGTGATG	CCAACCAAGAATTTCCGAACAGT	ChIP
Snr6 Upstream	-173 to -94	GTCATCTTCCTGGACCTCATG	GCAATGAAACTCTAAAGTATCATCGATTCAG	ChIP
Snr6 Promoter	-118 to +10	CGATGATACTTTAGAGTTTCATTGC	CTTCGCGAACACATAGTTGC	ChIP
Snr6 Gene	+63 to +189	GTTCCCCTGCATAAGGATGAACCG	GGAAGATAAAGATACACTGCTG	ChIP
Rpr1 Upstream	-281 to -175	CGTGTGTTTTATATGTCTCTTATCTAAG	GGTCGTTAACATATCCTGTTG	ChIP
Rpr1 Gene	+41 to +134	CAGAAGGATCCCCACCTATG	CGACATTAACCCGGAGGAC	ChIP/ RNA
Rpr1 3'-end	+365 to +486	CCAGCCCATATCCAACTTCCA	CCATATCTAATCTAACCAAATCCAAAC	ChIP
SCR1 Upstream **A**	-398 to -203	CATTCCGCAGTACCATGTCAAGAAC	GTCAGATTGTCACAAGCTATGGATGTC	ChIP
SCR1 Promoter **B**	-191 to +4	CAGTCTTATTTGATGATTCACACATTCCTTG	GCCTAGCACAATTGGAATAAACTTTCG	ChIP

SCR1 5'-end **C**	+5 to +181	TGTAATGGCTTTCTGGTGGGATG	AGCTCTGCCCAGGACAAATTTAC	ChIP/ RNA
SCR1 Middle **D**	+160 to +361	TAAATTTGTCCTGGGCAGAGCTGT	TGACGCTGGATAAAACTCCCCTAA	ChIP
SCR1 3'-end **E**	+393 to +562	ATAGCACATATCAGTCGGATAATT	ATCCCTCATTGGGACGGTTAATTAG	ChIP
SCR1 Downstream **F**	+541 to +728	ATTAACCGTCCCAATGAGGGATGT	TTATCCTTCTTATATTCCGCTGCCG	ChIP
tK(UUU)G2	+51 to +144	AAGCAAGGATACCGAAATGTCAGG	GGACATTCCATTGATGGCTATTGTAG	ChIP
tL(CAA)G1	+1 to +155	GGTTGTTTGGCCGAGCGGTCTA	CGCGGGTAGCTTGATCACAGAAC	ChIP
SNR52	-1 to +112	TCTACTATGATGAATGACATTAGCGTG	CAATAGTGACAAAAAATAAATTTCAGAAGGAAGG	ChIP
snR6	+10 to +109	GTAACCCTTCGTGGACATTTGG	ACGAAATAAATCTCTTTGTAAAACG	ChIP/ RNA
tY(GUA)J2	+1 to +86	CTCTCGGTAGCCAAGTTGGTTTAAGGCG	CCCGGGGGCGAGTCGAACGCCCGA	ChIP/ RNA
tK(UUU)G1	+26 to +93	GTTCGGCTTTTAAGCGCATT	CTCATAGGGGGCTCGAA	RNA
tP(UGG)O3*	+36 to +104	GCGATCCCCCTGATCAAA	GGGCGAGCTGGGAATT	RNA
tM(CAU)C	+14 to +72	AGTGGAAGCGCGCAG	TAGCGCCGCTCGGTTT	RNA
tG(CCC)D	+1 to +72	GCGAAAGTGGTTCAGTG	TGCGGAAGCCGGGAA	RNA
snR52	+1 to +91	TACTATGATGAATGACATTAGCGTGAACA	TTCAGAAGGAAGGCAACATAAGTTTT	RNA

* marks the primers unique for the isogene.
