# Supplementary material for: Genome-Wide Mapping of Yeast Histone Chaperone Anti-Silencing Function 1 Reveals Its Role in Condensin Binding with Chromatin
Source: PLoS One. 2014 Sep 29;9(9):e108652. doi: 10.1371/journal.pone.0108652 (PMC4181348; doi:10.1371/journal.pone.0108652)
Supplement: Table S3 — Different categories of the genome-wide chromosomal features targeted by Asf1. (PDF) [file pone.0108652.s007.pdf]

**Table S3: Different categories of genome-wide chromosomal features targeted by Asf1.** Numbers in the column 2 indicate the total number of features occupied by Asf1 in that category. Peaks were called using HOMER package, the statistical significance for the same is given as p-value range from the highest to the lowest for all targets in the category. Out of the 32 telomeres, Asf1 occupancy could be ascertained only on 10 telomeres.

| **Feature category** | **No. of Asf1 targets** | **% of Asf1 targets** | **p-value range** |
|---|---|---|---|
| ARS | 13 | 2.2 | 5.87E-19 to 0.00E+00 |
| Centromeres | 16 | 2.7 | 4.94E-322 to 0.00E+00 |
| Telomeres | 10 | 1.7 | 5.65E-16 to 7.05E-288 |
| Other small RNAs | 8 | 1.4 | 2.66E-14 to 0.00E+00 |
| snoRNAs | 39 | 6.6 | 6.10E-14 to 8.25E-208 |
| tRNAs | 262 | 44.4 | 4.94E-324 to 0.00E+00 |
| ORFs | 242 | 41.0 | 6.10E-14 to 0.00E+00 |
