# Supplementary material for: Genome-Wide Mapping of Yeast Histone Chaperone Anti-Silencing Function 1 Reveals Its Role in Condensin Binding with Chromatin
Source: PLoS One. 2014 Sep 29;9(9):e108652. doi: 10.1371/journal.pone.0108652 (PMC4181348; doi:10.1371/journal.pone.0108652)
Supplement: Table S4 — Functional distribution of Asf1-occupied 242 pol II genes. (PDF) [file pone.0108652.s008.pdf]

Table S4: Functional distribution of Asf1-occupied 242 pol II genes

Functional Category	Genome Matches	Asf1 Occupied	Overlap p-value
Carbohydrate metabolism	505	29	5.60e-03
Energy	367	20	3.34e-02
Transcription	1077	30	9.41e-01
Ribosome biogenesis	310	61	1.34e-30
Translation elongation	21	6	6.22e-05
Nucleic acid binding	341	21	8.47e-03
Cellular transport	1038	23	9.97e-01
Stress response	450	16	5.24e-01
Cell death	19	3	2.76e-02
Cell wall biogenesis	214	12	7.43e-02

MIPS Functional Catalogue annotation (http://mips.helmholtz-muenchen.de/proj/funcatDB/) was used to look for the functional categories having significant p-Value. Most significant enrichment of Asf1 is found on the genes coding for ribosome biogenesis.

To correct for multiple comparisons in multiple hypothesis testing for the 845 taxonomical allowed FunCats, we calculated the Bonferoni correction as well as the False discovery rate (FDR) control with an experiment-wide significance level alpha of 0.05. The table is sorted by the p-value. The additional index of k B=Bonferoni correction < 0.05, F:p-value < False discovery rate and *:p-value < 0.05 was used.
