# Supplementary material for: Genome-Wide Mapping of Yeast Histone Chaperone Anti-Silencing Function 1 Reveals Its Role in Condensin Binding with Chromatin
Source: PLoS One. 2014 Sep 29;9(9):e108652. doi: 10.1371/journal.pone.0108652 (PMC4181348; doi:10.1371/journal.pone.0108652)
Supplement: Methods S1 — This file contains details of the methods followed and the supplementary references cited in the Supporting Information files. (PDF) [file pone.0108652.s010.pdf]

This document gives supplementary methods and references.

## I. SUPPLEMENTARY METHODS

### ChIP-Seq sample preparation and data analysis

ChIP-seq data analysis for Asf1 occupancy was done essentially as described in detail earlier [41]. Single-end 36 bp sequencing reads, obtained from Illumina Genome Analyzer II, were first preprocessed using FASTX-Toolkit (http://hannonlab.cshl.edu/fastx_toolkit/index.html). High-quality sequencing reads (phred score >20; with a minimum 80% of the bases having quality score 20 or higher) were selected for further analysis and aligned, reporting unique and best alignment for each read, to budding yeast genome version sacCer 3. We used BOWTIE tool version 0.12.8 (http://bowtie-bio.sourceforge.net/index.shtml/) [56] for read alignment to the recent assembly of the budding yeast genome (SacCer_Apr2011/sacCer3 version). For read alignment to the genome, only unique reads with the best alignment were chosen which gave very high genome-coverage (>100X) for the two biological replicates. The Pearson correlation coefficient (r) between the two biological replicates was found to be 0.983 indicative of high reproducibility of the ChIP-seq experiment. In order to visualize tag-density across the yeast chromosomes on UCSC browser, ready-to-visualize bedgraph files were created using HOMER package v3.13 (http://biowhat.ucsd.edu/homer/chipseq/) [57]. Briefly, aligned reads were extended to the average fragment size (~160 bp) and read coverage on each base across the genome was calculated. Read coverage was then scaled to one million and normalized with the total number of reads. The reproducibility of the ChIP-seq experiment was ascertained by visualizing bedgraph files of the two replicates on UCSC genome browser, which showed identical distribution patterns. Peak finding was performed using HOMER to find centered peaks with a minimum size of 150 bp (1.5 fold-enrichment over mock sample, FDR threshold = 0.1%).

Yeast genome has ~60,000 nucleosomes [58]. Even with >100X deep genome-coverage, we found only 8,660 putative peaks (total 607 peaks after normalizing with mock sample) suggesting minimum 607 locations where Asf1 binds strongly that can be captured through ChIP-seq method. The correct assignment of

peaks was further confirmed by visualizing Asf1 tag distribution on UCSC genome browser and simultaneous comparison with the assigned peaks.

In order to view the association of Asf1 with different chromosomal features, heat-maps of the occupancy data covering 500 base pairs upstream and downstream of each feature were created using Multiexperiment Viewer (MeV, v4.8.1) [59]. Asf1 occupancy within 1000 bp upstream and downstream regions of a reference point on each chromosomal feature was calculated taking 10 bp window/bin and 2 bp overlap. Asf1 occupancy data were compared with previously published details of genome-wide occupancy profiles of pol II, nucleosomes, pol III and condensin. Smoothened Pol II bedgraph file [33] and high-resolution nucleosome occupancy data [46] were used to calculate average profile of pol II and nucleosome, respectively, at five categories of genes. For comparison with Brn1 data, average occupancy of Brn1 was calculated for each assigned peak of Asf1 using previously published data [47].

**RNA estimation**

Total RNA was isolated from wild-type and *asf1*Δ cells using hot phenol method. Briefly, extracted RNA was treated with DNase I (NEB) for 30 min at 37 $^{0}$C and poly-adenylated using poly(A)-polymerase [41] after inactivating DNase I at 65 $^{0}$C for 10 min. 5 μg of poly-adenylated RNA was used to make the cDNA, which was used for qPCR in LC480 (Roche) Real Time PCR machine. *SCR1* was used as an internal control [28]. Gene-expression microarray data from the previous reports were used to find pol II-transcribed genes, which are misregulated in *asf1*Δ cells [25,28].

**Histone Exchange Assay**

Yeast strain YYK9 [60] and isogenic *asf1*Δ cells were transformed with plasmid pYES3/CT H3-3HA that contains *GAL1*-driven H3-$(HA)_3$ gene for histone exchange assay [34]. Cells were grown in raffinose-supplemented synthetic dropout $Trp^-$ medium to $OD_{600}$ of 0.5. Cells were then arrested in G1-phase (to eliminate the contribution of replication-coupled histone exchange) by treatment with yeast α-mating factor (Sigma-Aldrich) for 3 h and the expression of tagged H3 was induced with galactose for varying time-intervals. Tagged H3 was immuno-precipitated with

anti-HA antibody (Millipore) and ChIP DNA was measured using qPCR at varying time intervals. A progressive increase in tagged H3 occupancy was taken as an indication of active H3/H4 exchange.

**Nucleosome occupancy measurement**

In order to quantify the nucleosome density in the coding region of pol III-transcribed genes, yeast cells were grown to OD 0.6 and fixed with formaldehyde for 10 min. Excess formaldehyde was quenched by adding glycine and cells were harvested. Cell pellet was washed twice with ice cold 1XPBS. Spheroplasts were made using zymolyase and lysed in 1X NPS buffer (10 mM Tris-Cl pH 7.5, 100 mM NaCl, 5 mM $MgCl_2$, 2 mM $CaCl_2$, 1 mM β-mercaptoethanol, 0.5 mM Spermidine, 0.075% NP-40). Chromatin was digested using MNase to give major mononucleosomal DNA and a small fraction dinucleosomal DNA. Digested chromatin was de-crosslinked, phenol extracted and purified. To obtain mononucleosmal DNA, isolated DNA was run into 2% agarose gel and the mononucleosomal DNA-band was excised and purified using spin column. Purified mononucleosomal DNA was used for qPCR measurements. Ct value for similarly prepared, naked genomic DNA was measured for normalization. Nucleosome occupancy was measured at pol III-transcribed genes against an ORF-free region on chromosome V (control).

**Brn1 ChIP and Western blotting**

Wild-type and *asf1*Δ cells expressing sole copy of Brn1-GFP under its native promoter [61] were used for Brn1 ChIP measurements. ChIP samples were prepared as for Asf1, except that cross-linking was done for 3 h. Average values from four independent experiments with standard deviation (bars) are shown in the Figure 5B. Values represent fold-enrichment over *COX3* region. For total Brn1-GFP levels estimations, yeast cells were grown to OD 0.6, collected by centrifugation and flash frozen in liquid nitrogen. Next, whole-cell extracts were prepared using TCA method, proteins were resolved on SDS-PAGE gels and probed with anti-GFP (Brn1) or anti-GAPDH antibodies.

## II. SUPPLEMENTARY REFERENCES

[56] Langmead B, Trapnell C, Pop M, Salzberg SL (2009) Ultrafast and memory-efficient alignment of short DNA sequences to the human genome. Genome biology 10: R25.

[57] Heinz S, Benner C, Spann N, Bertolino E, Lin YC, Laslo P, Cheng JX, Murre C, Singh H, Glass CK (2010) Simple combinations of lineage-determining transcription factors prime cis-regulatory elements required for macrophage and B cell identities. Mol Cell 38, 576-589.

[58] Mavrich TN, Ioshikhes IP, Venters BJ, Jiang C, Tomsho LP, Qi J, Schuster SC, Albert I, Pugh BF (2008) A barrier nucleosome model for statistical positioning of nucleosomes throughout the yeast genome. Genome research 18, 1073-1083.

[59] Saeed AI, Sharov V, White J, Li J, Liang W, Bhagabati N, Braisted J, Klapa M, Currier T, Thiagarajan M et al. (2003) TM4: a free, open-source system for microarray data management and analysis. BioTechniques 34, 374-378.

[60] Kamimura Y, Tak YS, Sugino A, Araki H (2001) Sld3, which interacts with Cdc45 (Sld4), functions for chromosomal DNA replication in Saccharomyces cerevisiae. The EMBO Journal 20, 2097-2107.

[61] Varela E, Shimada K, Laroche T, Leroy D, Gasser SM (2009) Lte1, Cdc14 and MEN-controlled Cdk inactivation in yeast coordinate rDNA decompaction with late telophase progression. EMBO J 28: 1562-1575.

[62] Ng HH, Xu RM, Zhang Y, Struhl K (2002) Ubiquitination of histone H2B by Rad6 is required for efficient Dot1-mediated methylation of histone H3 lysine 79. J. Biol. Chem. 277, 34655-34657.
